# Supplementary material for: Older adults are relatively more susceptible to impulsive social influence than young adults
Source: Commun Psychol. 2024 Sep 23;2:87. doi: 10.1038/s44271-024-00134-0 (PMC11420232; doi:10.1038/s44271-024-00134-0)
Supplement: Supplementary file 2 — Supplementary Information [file 44271_2024_134_MOESM2_ESM.pdf]

Supplementary Information

Supplementary Methods

Posterior predictive checks

We used posterior predictive checks to evaluate the extent to which the posterior estimation of our winning model accurately replicates the key features of participant behaviour (e.g., their performances of learning about others' preferences). For this purpose, we used a post-hoc absolute-fit approach[1], which factored in participants' actual decisions and option pairs, to generate predictions using the entire set of posterior Markov chain Monte Carlo (MCMC) samples of the winning model. To be specific, we let the winning model generate synthetic decisions repeatedly, matching the number of MCMC samples (i.e., 8000 times) for each trial and each participant, with the individual-level posterior parameters obtained through model estimation. Then, we analysed the synthetic data using the same methods as those applied to the actual data, using a linear mixed-effects model (LMM). This LMM incorporated fixed effects of age group (*older* vs *young*), other's preference (*patient* vs *impulsive*), and their interaction, along with a random subject-level intercept.

Model-free index analysis

We also used another model-free index, the choice proportions of delayed options, to gauge participants' temporal preferences and to examine whether we can replicate preference-shifting patterns observed using our winning model. We calculated the choice proportions of delayed options in each *Self* block (baseline, after learning the Impulsive Other, and after learning the Patient Other) and determined the differences between the baseline block and the post-learning blocks. Higher proportions of choosing delayed options indicate more patient temporal preferences. Positive values of the differences in choice proportions of delayed options indicate participants became more patient after learning others' preferences, while negative values indicate participants became more impulsive. To facilitate comparison with the original results, we flipped the data points after learning the Impulsive Other. This means that positive values in the differences in choice proportions of delayed options always indicate participants' preferences became more similar to those of the others. We analysed the differences in choice proportions of delayed options using the same method we applied to signed KL divergence (i.e., the model-based proxy of susceptibility to social influence). Specifically, we used a LMM that included fixed effects of age group (*older* vs *young*), other's preference (*patient* vs *impulsive*), and their interaction, along with a random subject-level intercept.

Statistical analysis

Linear mixed-effects models (LMMs) were used to predict participants' learning accuracy, signed KL divergence, and task-specific questionnaires. These models included fixed effects of age group (*older* vs *young*), other's preference (*patient* vs *impulsive*), and their interaction, along with a random subject-level intercept. An additional analysis of signed KL divergence also incorporated participants' baseline temporal impulsivity (km; continuous covariates, centred around the grand mean) and its interaction with age groups and other's preferences (including the three-way interaction) as fixed terms. In addition, control analyses of signed KL divergence included the standardised IQ scores from the WTAR as a fixed term, without interacting with the other terms (age group, other's preference, or self baseline impulsivity). A further control analysis was performed to examine the effect of the order of others' preferences on the signed KL divergence.

We examined another two LMMs that included an additional fixed effect of the order of other's preferences (*patient first* vs *impulsive first*) without interacting with other terms, as a control variable.

The linear mixed-effects models were set up as follows:
LMM1: Accuracy ~ Group * Preference + (1|ID)

LMM2: Questionnaires (confidence/similarity) ~ Group * Preference + (1|ID)

LMM3a: Signed KL divergence ~ Group * Preference + (1|ID)
LMM3b: Signed KL divergence ~ Group * Preference * Self baseline impulsivity + (1|ID)
LMM3c: Signed KL divergence ~ Group * Preference + IQ + (1|ID)
LMM3d: Signed KL divergence ~ Group * Preference * Self baseline impulsivity + IQ + (1|ID)
LMM3e: Signed KL divergence ~ Group * Preference + Order + (1|ID)
LMM3f: Signed KL divergence ~ Group * Preference * Self baseline impulsivity + Order + (1|ID)

Supplementary Note 1

Susceptibility to social influence does not depend on IQ, executive function, or the order of others' preferences

In a control analysis, we examined whether people's susceptibility to social influence depends on their general intelligence. We used a linear mixed-effects model that included fixed effects for age group, other's preference, and their interaction, along with a random subject-level intercept. It also included the standardised scores from the WTAR as a fixed term, without interacting with the other terms. Controlling for IQ did not change any of our results (main effect of IQ: b = -0.01, 95% CI = [-0.02 0.00], Z = -1.30, P = 0.19, BF_{01} = 5.03; significant main effect of age group: b = 0.48, 95% CI = [0.23 0.74], Z = 3.68, P < 0.001; significant main effect of other's preference: b = 0.58, 95% CI = [0.32, 0.84], Z = 4.38, P < 0.001; significant interaction between age group and other's preference: b = -0.57, 95% CI = [-0.94, -0.21], Z = -3.08, P = 0.002).

Controlling for the order of other's preferences did not change any of our main results, without (LMM3e: main effect of order of others: b = 0.07, 95% CI = [-0.11 0.25], Z = 0.75, P = 0.46, BF_{01} = 5.75; significant main effect of age group: b = 0.47, 95% CI = [0.22 0.73], Z = 3.6, P < 0.001; significant main effect of other's preference: b = 0.57, 95% CI = [0.31 0.83], Z = 4.33, P < 0.001; significant interaction between age group and other's preference: b = -0.56, 95% CI = [-0.93 -0.2], Z = -3.04, P = 0.002) or with (LMM3f: main effect of order of others: b = 0.07, 95% CI = [-0.11 0.26], Z = 0.79, P = 0.43, BF_{01} = 6.07; significant main effect of age group: b = 0.45, 95% CI = [0.19 0.71], Z = 3.38, P < 0.001; significant main effect of other's preference: b = 0.54, 95% CI = [0.28 0.81], Z = 3.99, P < 0.001; significant interaction between age group and other's preference: b = -0.53, 95% CI = [-0.91 -0.16], Z = -2.78, P = 0.005) self baseline impulsivity as covariates.

Table S1. *Correlations between impulsive signed D_{KL} and the factor 'Affective empathy & emotional motivation' scores for older adults, controlling for general intelligence and executive function*

	Impulsive signed D_{KL} in older adults			
	Standard	Partial		
		IQ	Attention	Memory
$r_{s(71)}$	0.29 [0.06 0.48]	0.29 [0.07 0.49]	0.28 [0.06 0.48]	0.26 [0.04 0.47]
P	0.014*	0.011*	0.015*	0.024*

Note. r_s: Spearman's Rho correlation coefficients; 95% confidence intervals are indicated in square brackets. *P*: *p*-values of correlations.

Table S2. *Correlations between patient signed D_{KL} and the factor 'Autistic & alexithymic traits' scores for older adults, controlling for general intelligence and executive function*

	Patient signed D_{KL} in older adults			
	Standard	Partial		
		IQ	Attention	Memory
$r_{s(66)}$	0.34 [0.11 0.54]	0.34 [0.11 0.54]	0.34 [0.11 0.53]	0.35 [0.12 0.54]
P	0.004**	0.004**	0.005**	0.004**

Note. r_s: Spearman's Rho correlation coefficients; 95% confidence intervals are indicated in square brackets. *P*: *p*-values of correlations.

Table S3. *Model parameters from the winning model for each experimental block*

	Young		Older	
	km	*ku*	*km*	*ku*
Self baseline	-4.79 [0.22]	1.37 [0.06]	-5.16 [0.25]	1.47 [0.06]
Impulsive Other	-3.34 [0.25]	1.77 [0.06]	-3.65 [0.21]	1.63 [0.04]
Self after Impulsive Other	-4.87 [0.26]	1.31 [0.06]	-4.76 [0.24]	1.18 [0.04]
Patient Other	-6.58 [0.17]	1.93 [0.07]	-6.19 [0.15]	1.65 [0.03]
Self after Patient Other	-5.48 [0.22]	1.37 [0.07]	-5.49 [0.21]	1.18 [0.04]

Note. *km*: mean of discounting distribution; *ku*: standard deviation of discounting distribution. The numbers shown are group means [standard errors].

Table S4. *Correlations between learning performances and signed KL divergence (D_{KL})*

	Young		Older	
	Impulsive	Patient	Impulsive	Patient
r_s	$r_{s(66)}$ = -0.13 [-0.36 0.11]	$r_{s(70)}$ = 0.07 [-0.16 0.30]	$r_{s(72)}$ = 0.06 [-0.17 0.28]	$r_{s(66)}$ = 0.06 [-0.18 0.29]
P	0.274	0.540	0.637	0.625
BF_{01}	4.46	6.74	6.12	5.39

Note. r_s: Spearman's Rho correlation coefficients; 95% confidence intervals are indicated in square brackets. *P*: *p*-values of correlations. BF_{01} indicates the strength of evidence for the null hypothesis.

Table S5. *LMM predicting unsigned differences in choice proportions of later-and-larger (LL) options.*

**Fixed effect**	***beta***	**95% CI**	***Z***	***P***
(Intercept)	-0.47	[-2.63 1.69]	-0.43	0.67
Group (*older* vs *young*)	4.70	[1.70 7.68]	3.07	0.002
Others (*patient* vs *impulsive*)	5.80	[2.79 8.82]	3.78	<0.001
Group x Others	-7.70	[-11.91 -3.42]	-3.54	<0.001

Note. LMM: linear mixed-effects model; 95% CI: 95% confidence interval.

Table S6. *LMM predicting susceptibility to social influence, after removing outliers*

**Fixed effect**	***beta***	**95% CI**	***Z***	***P***
(Intercept)	0.01	[-0.14 0.16]	0.12	0.90
Group (*older* vs *young*)	0.33	[0.12 0.55]	3.09	0.002
Others (*patient* vs *impulsive*)	0.45	[0.24 0.66]	4.15	<0.001
Group x Others	-0.42	[-0.72 -0.12]	-2.76	0.006

Note. LMM: linear mixed-effects model; 95% CI: 95% confidence interval.

Table S7. *LMM predicting susceptibility to social influence, with self baseline temporal impulsivity as covariates (centred around the grand mean), after removing outliers*

**Fixed effect**	***beta***	**95% CI**	***Z***	***P***
(Intercept)	0.00	[-0.15 0.16]	0.06	0.96
Group (*older* vs *young*)	0.31	[0.10 0.53]	2.91	0.004
Others (*patient* vs *impulsive*)	0.44	[0.23 0.66]	4.01	<0.001
Self baseline *km*	0.01	[-0.07 0.10]	0.32	0.75
Group x Others	-0.40	[-0.71 -0.09]	-2.57	0.01
Group x Self baseline *km*	-0.11	[-0.23 0.00]	-2.02	0.04
Others x Self baseline *km*	0.02	[-0.11 0.15]	0.24	0.81
Group x Others x Self baseline *km*	0.10	[-0.01 0.29]	1.13	0.26

Note. LMM: linear mixed-effects model; 95% CI: 95% confidence interval; *km*: the estimated mean of discounting distribution from the winning model.

Table S8. *Correlations between the factors and signed KL divergence (D_{KL})*

			Autistic & alexithymic traits	Psychopathic traits	Affective empathy & emotional motivation
Young	Impulsive	$r_{s(66)}$	0.11 [-0.13 0.34]	0.01 [-0.23 0.25]	-0.13 [-0.36 0.11]
	D_{KL}	BF_{01}	4.90	6.85	5.33
	Patient	$r_{s(69)}$	-0.04 [-0.27 0.20]	-0.07 [-0.30 0.17]	-0.06 [-0.29 0.18]
	D_{KL}	BF_{01}	7.15	6.40	6.48
Older	Impulsive	$r_{s(71)}$	-0.07 [-0.30 0.16]	0.03 [-0.20 0.26]	**0.29 [0.06 0.48]***
	D_{KL}	BF_{01}	6.59	6.51	0.25
	Patient	$r_{s(66)}$	**0.34 [0.11 0.54]****	0.11 [-0.13 0.34]	-0.11 [0.34 0.13]
	D_{KL}	BF_{01}	0.22	5.22	3.25

Note. $r_{s(df)}$: Spearman's Rho correlation coefficients with degrees of freedom; 95% confidence intervals are indicated in square brackets. BF_{01} indicates the strength of evidence for the null hypothesis. *$P < 0.05$, **$P < 0.01$.

Table S9. *Between-group comparisons of the correlation coefficients between the factors and signed KL divergence (D_{KL})*

		Autistic & alexithymic traits	Psychopathic traits	Affective empathy & emotional motivation
Impulsive D_{KL}	*diff*	0.19 [-0.15 0.51]	-0.02 [-0.35 0.31]	-0.41 [-0.72 -0.08]
	Z	1.09	-0.13	-2.45
	P	0.276	0.898	**0.014***
	BF_{01}	4.99	5.14	1.56
Patient D_{KL}	*diff*	-0.38 [-0.68 -0.05]	-0.17 [-0.50 0.16]	0.06 [-0.28 0.38]
	Z	-2.27	-1.01	0.32
	P	**0.023***	0.312	0.746
	BF_{01}	2.08	2.43	3.25

Note. *diff*: differences in correlation coefficients between young and older groups; 95% confidence intervals are indicated in square brackets. BF_{01} indicates the strength of evidence for the null hypothesis. *$P < 0.05$.

Table S10. *Correlations between the factors and signed KL divergence (D_{KL}), after excluding outliers*

			Autistic & alexithymic traits	Psychopathic traits	Affective empathy & emotional motivation
Young	Impulsive	$r_{s(65)}$	0.09 [-0.15 0.33]	0.02 [-0.22 0.26]	-0.13 [-0.36 0.11]
	D_{KL}	BF_{01}	5.52	7.17	4.57
	Patient	$r_{s(68)}$	-0.03 [-0.26 0.21]	-0.07 [-0.30 0.17]	-0.09 [-0.31 0.15]
	D_{KL}	BF_{01}	7.46	6.37	6.22
Older	Impulsive	$r_{s(70)}$	-0.08 [-0.31 0.15]	0.05 [-0.18 0.28]	**0.28 [0.05 0.48]***
	D_{KL}	BF_{01}	6.06	5.29	0.34
	Patient	$r_{s(65)}$	**0.35 [0.12 0.55]****	0.16 [-0.09 0.38]	-0.11 [0.34 0.13]
	D_{KL}	BF_{01}	0.17	1.98	2.95

Note. $r_{s(df)}$: Spearman's Rho correlation coefficients with degrees of freedom; 95% confidence intervals are indicated in square brackets. BF_{01} indicates the strength of evidence for the null hypothesis. *$P < 0.05$, **$P < 0.01$.

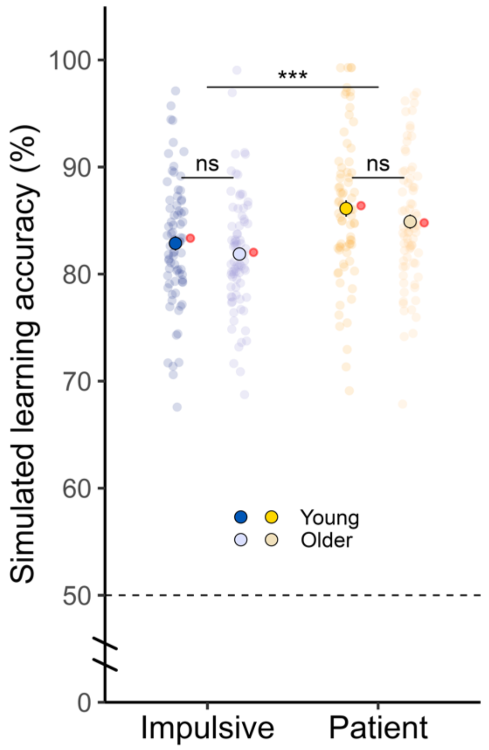

Figure S1. **Posterior predictive checks of the winning KU model.** Comparison of simulated learning accuracy shows no statistically significant difference in learning performance of the others' preferences between the two age groups (no main effect of age group: b = -0.01, 95% CI = [-0.03 0.01], Z = -1.04, P = 0.30, BF_{01} = 2.47). In addition, both simulated young (N = 76) and older (N = 78) participants demonstrated better learning of the preferences of patient others compared to impulsive ones (significant main effect of other's preference: b = 0.03, 95% CI = [0.02 0.05], Z = 4.35, P < 0.001). Large circles with border lines indicate the mean, error bars represent the standard error of the mean, dots show raw data, and asterisks denote the significant main effect of other's preference based on the linear mixed-effects model. Note that the axis includes a discontinuity between 0% and 50% to highlight the range of observed data more clearly. The dashed line at 50% indicates the chance level of performance. *** P < 0.001; ns: not significant. Red dots are the means of actual data.

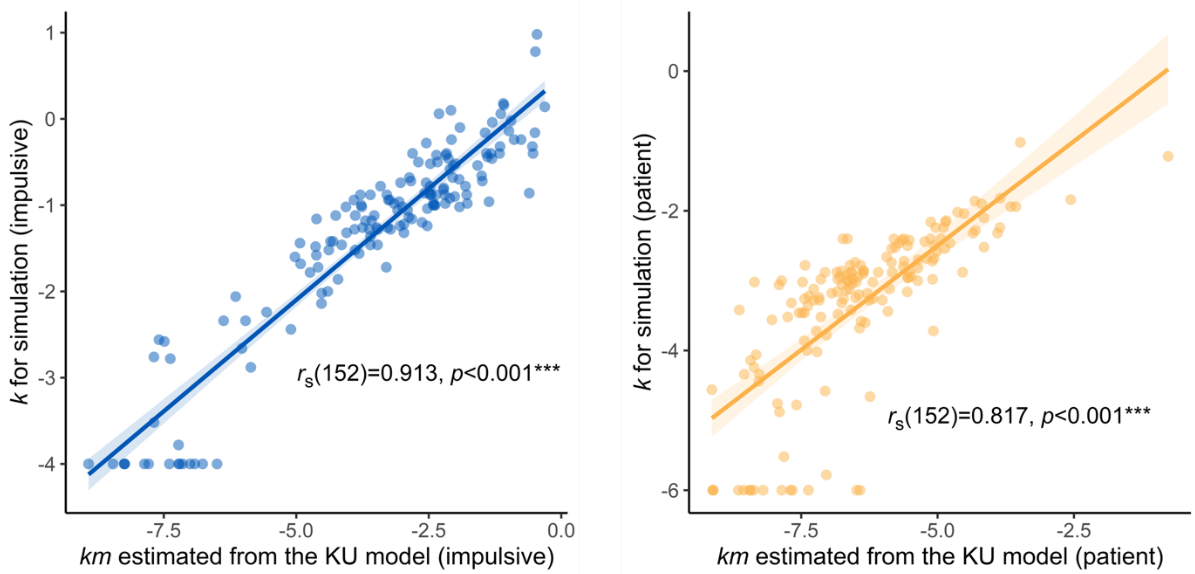

Figure S2. **Correlations between parameters estimated from the winning model and those used to simulate others' choices.** To assess our winning model's accuracy in capturing the learning process, we analysed the correlations between the means of discounting distribution (*km*) from the Bayesian winning model fitted with experimental data and the hyperbolic discount rates (*k*) from the non-Bayesian preference-temperature model that was used to simulate other's choices as task stimuli. The results showed a significant correlation in both conditions, for both impulsive (N = 154, $r_{s(152)}$ = 0.91 [0.88 0.94], P < 0.001) and patient (N = 154 $r_{s(152)}$ = 0.82 [0.76 0.86], P < 0.001) others. This suggests that our model accurately reflects the learning process.

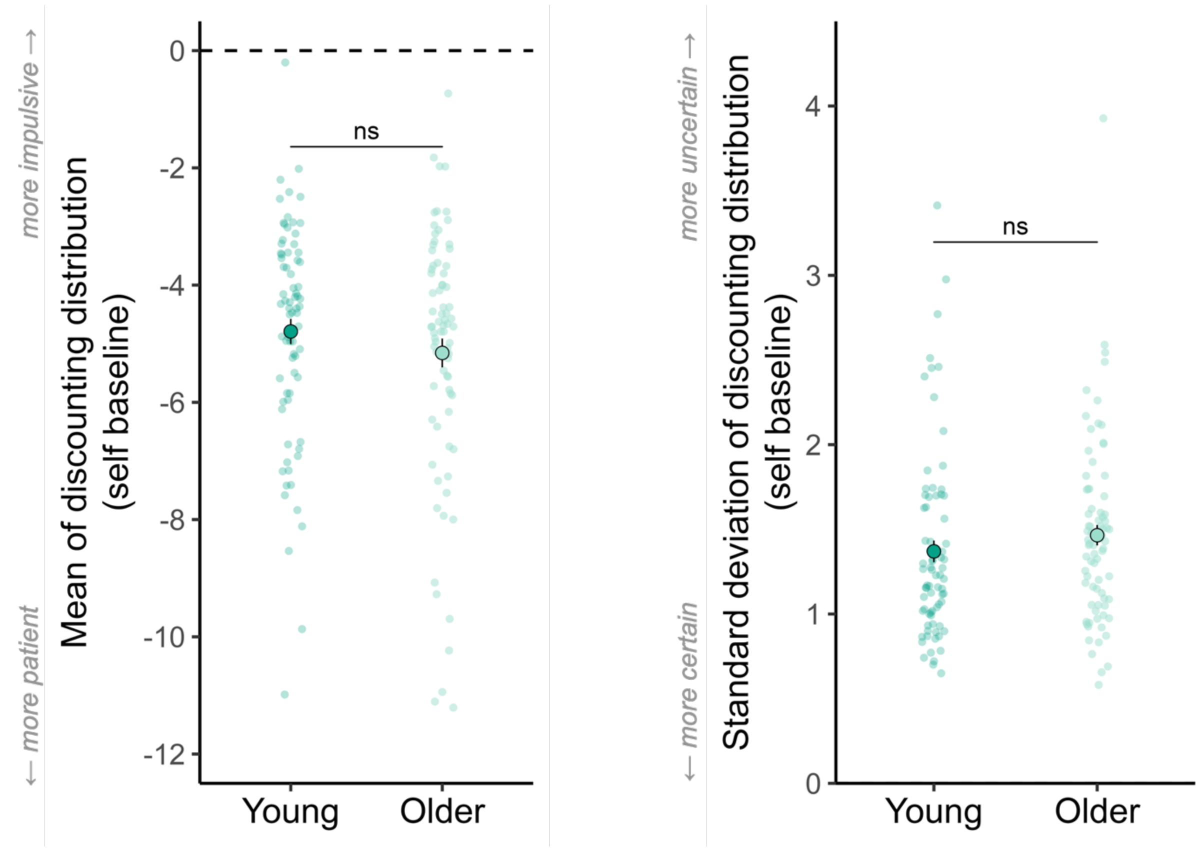

Figure S3. **Baseline temporal impulsivity and preference uncertainty do not significantly differ with age.** No statistically significant differences were noted in either the mean (independent Wilcoxon signed-rank test: W = 3243, Z = -1.01, $r_{(152)}$ = 0.08 [0.005 0.24], P = 0.314, BF_{01} = 3.47) or the standard deviation (W = 2481, Z = -1.74, $r_{(152)}$ = 0.14 [0.009 0.31], P = 0.081, BF_{01} = 2.31) of the discounting distribution at the baseline between two age groups (N = 76 for the young group and N = 78 for the older group).

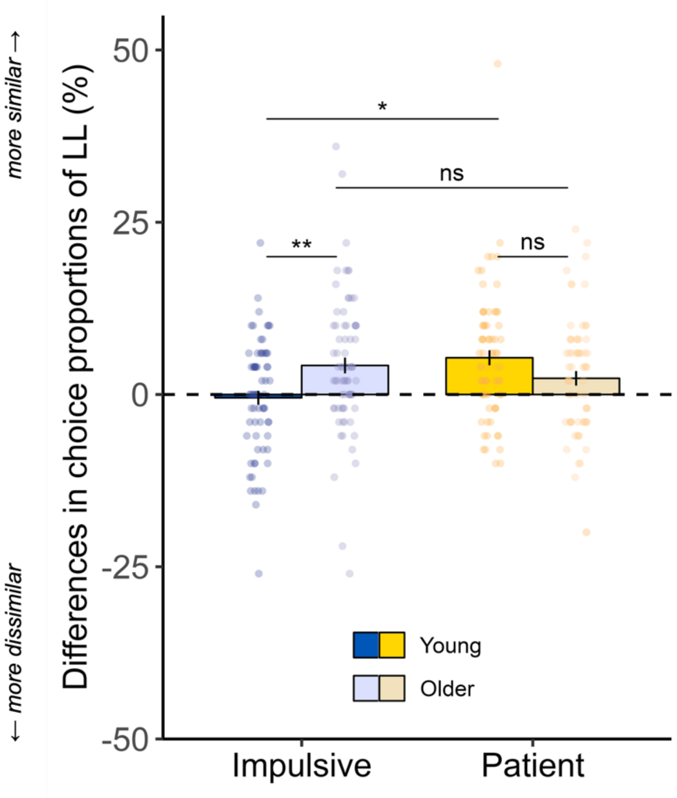

Figure S4. **Susceptibility to social influence quantified by model-free index, choice proportions of larger-and-later (LL) options.** The model-free index of temporal preferences and susceptibility to social influence produced (i.e., choice proportions of delayed options) results that were consistent with the model-based measures (i.e., signed KL divergence). Compared to young adults, older adults were more susceptible to impulsive social influence (W = 1823, Z = -2.83, $r_{(140)}$ = 0.24 [0.08 0.40], P = 0.005). Conversely, older and young adults were equivalently influenced by patient others (W = 2866, Z = -1.74, $r_{(138)}$ = 0.15 [0.01 0.31], P = 0.082, BF_{01} = 1.22). Sample sizes differ across conditions due to the unavailability of relevant data for some participants (N = 68 for young impulsive, N = 72 for young patient, N = 74 for older impulsive, and N = 68 for older patient). Bars show group means, error bars are standard errors of the mean, dots are raw data, and asterisks represent significant two-sided between-group and within-group nonparametric t tests. * P < 0.05; ** P < 0.01; ns: not significant. The data points after learning the Impulsive Other were flipped to facilitate comparison with the original results.

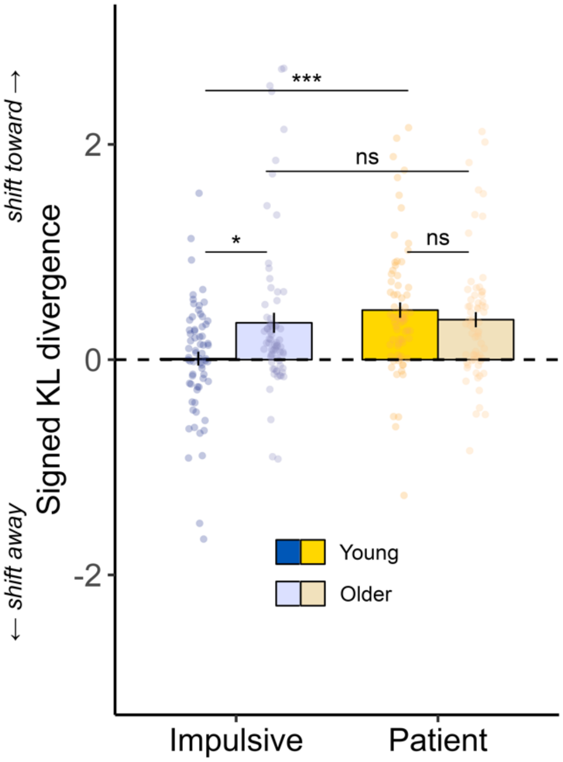

Figure S5. **Susceptibility to social influence quantified by the signed KL divergence (*D_{KL}*) after removing outliers.** After removing outliers (i.e., any data points falling below or above three standard deviations from the group mean, all our main results stayed the same. Older adults were more susceptible to impulsive social influence than young adults (W = 1861, Z = -2.44, $r_{(138)}$ = 0.21 [0.05 0.36], P = 0.015). Conversely, both older and young adults exhibited comparable susceptibility to patient social influence (W = 2655, Z = -1.17, $r_{(136)}$ = 0.10 [0.00 0.27], P = 0.24, BF_{01} = 3.20). Sample sizes differ across conditions due to the unavailability of relevant data for some participants (N = 67 for young impulsive, N = 71 for young patient, N = 73 for older impulsive, and N = 67 for older patient). Bars show group means, error bars are standard errors of the mean, dots are raw data, and asterisks represent significant two-sided between-group and within-group nonparametric *t* tests. * $P < 0.05$; *** $P < 0.001$; ns: not significant.

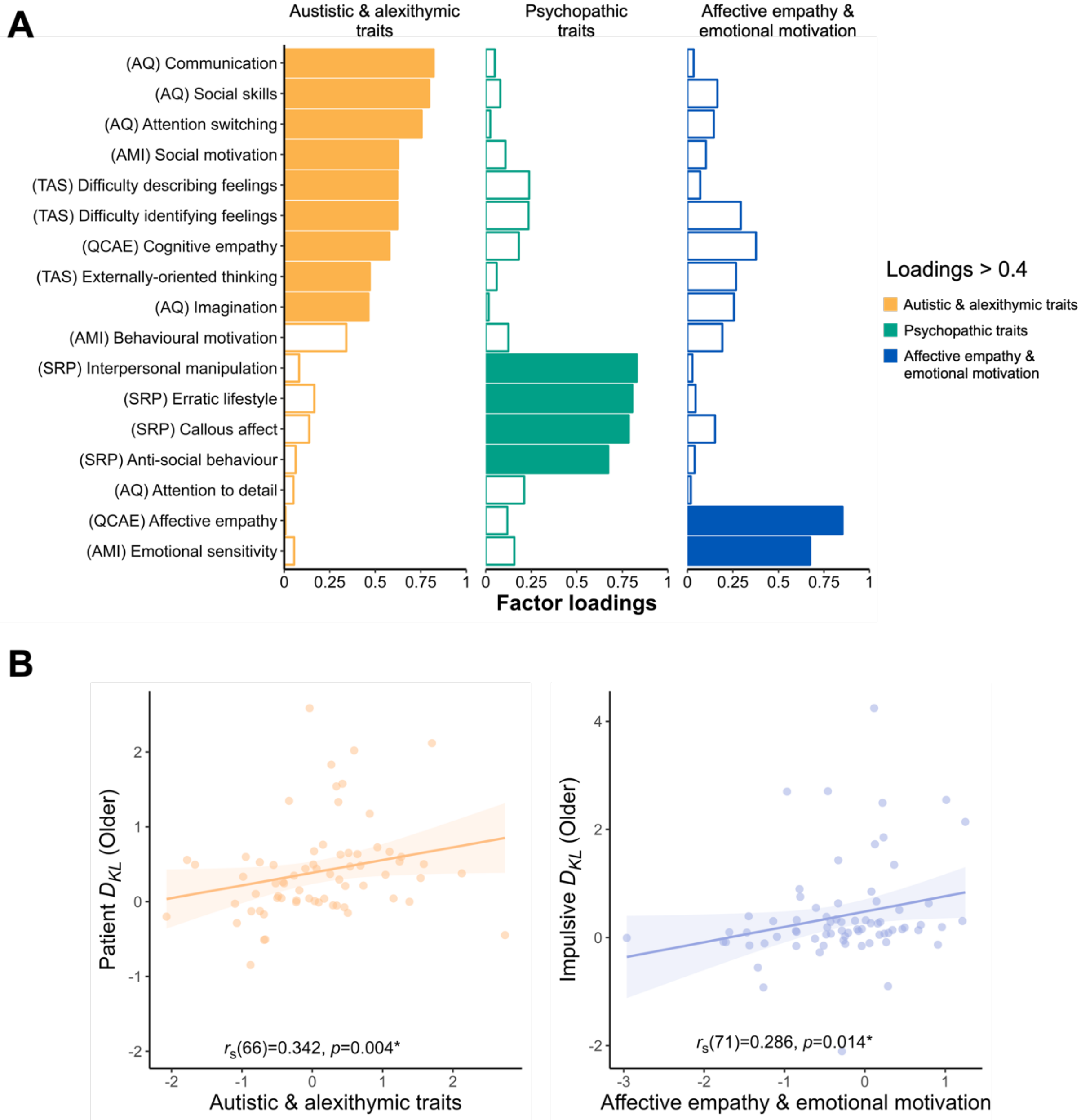

Figure S6. **Individual variations in susceptibility to social influence amongst older adults are related to socio-affective traits.** (A) Factor loadings for each subscale of the questionnaires completed. Exploratory factor analysis uncovered three distinct dimensions related to social affective cognition and behaviour: autistic & alexithymic traits, psychopathic traits, and affective empathy & emotional motivation. Note: the loadings displayed here are absolute values. AQ: Autism Quotient; AMI: Apathy-Motivation Index; TAS: Toronto Alexithymia Scale; QCAE: Questionnaire of Cognitive and Affective Empathy; SRP: Self-Report Psychopathy scale. (B) The factors 'Autistic & alexithymic traits' and 'Affective empathy & emotional motivation' related to susceptibility to social influence in older adults, depending on the nature of social influence. Older people with higher levels of autistic and alexithymic traits are more susceptible to patient social influence (N = 68), while those who

are more affectively empathetic and emotionally motivated display greater susceptibility to impulsive social influence (N = 73).

References

1. Zhang, L., Lengersdorff, L., Mikus, N., Gläscher, J. & Lamm, C. Using reinforcement learning models in social neuroscience: frameworks, pitfalls and suggestions of best practices. *Soc. Cogn. Affect. Neurosci.* **15**, 695–707 (2020).
