# Supplementary material for: Older adults are relatively more susceptible to impulsive social influence than young adults
Source: Commun Psychol. 2024 Sep 23;2:87. doi: 10.1038/s44271-024-00134-0 (PMC11420232; doi:10.1038/s44271-024-00134-0)
Supplement: Supplementary file 3 — Reporting Summary [file 44271_2024_134_MOESM3_ESM.pdf]

Corresponding author(s): Zhilin Su, Prof Patricia Lockwood

Last updated by author(s): Aug 19, 2024

# Reporting Summary

Nature Portfolio wishes to improve the reproducibility of the work that we publish. This form provides structure for consistency and transparency in reporting. For further information on Nature Portfolio policies, see our Editorial Policies and the Editorial Policy Checklist.

## Statistics

For all statistical analyses, confirm that the following items are present in the figure legend, table legend, main text, or Methods section.

| n/a | Confirmed | |
|---|---|---|
| ☐ | ☒ | The exact sample size ($n$) for each experimental group/condition, given as a discrete number and unit of measurement |
| ☐ | ☒ | A statement on whether measurements were taken from distinct samples or whether the same sample was measured repeatedly |
| ☐ | ☒ | The statistical test(s) used AND whether they are one- or two-sided<br>*Only common tests should be described solely by name; describe more complex techniques in the Methods section.* |
| ☐ | ☒ | A description of all covariates tested |
| ☐ | ☒ | A description of any assumptions or corrections, such as tests of normality and adjustment for multiple comparisons |
| ☐ | ☒ | A full description of the statistical parameters including central tendency (e.g. means) or other basic estimates (e.g. regression coefficient) AND variation (e.g. standard deviation) or associated estimates of uncertainty (e.g. confidence intervals) |
| ☐ | ☒ | For null hypothesis testing, the test statistic (e.g. $F$, $t$, $r$) with confidence intervals, effect sizes, degrees of freedom and $P$ value noted<br>*Give P values as exact values whenever suitable.* |
| ☐ | ☒ | For Bayesian analysis, information on the choice of priors and Markov chain Monte Carlo settings |
| ☐ | ☒ | For hierarchical and complex designs, identification of the appropriate level for tests and full reporting of outcomes |
| ☐ | ☒ | Estimates of effect sizes (e.g. Cohen's $d$, Pearson's $r$), indicating how they were calculated |

*Our web collection on statistics for biologists contains articles on many of the points above.*

## Software and code

Policy information about availability of computer code

| | |
|---|---|
| Data collection | MATLAB 2012a (The MathWorks Inc) with the Cogent 2000 v125 graphic toolbox (software developed by the University College London; used to be available at www.vislab.ucl.ac.uk/Cogent/). |
| Data analysis | Custom scripts in R v4.2.1 (with R Studio; including packages: lme4 v1.1-33, rstatix v0.7.1, Hmisc v4.7-2, psych v2.4.3, cocor v1.1-4, BayesFactor v0.9.12-4.4, BFpack v1.2.3, RStan v2.21.7, ggplot2 v3.4.2) and Stan v2.32. Data and code are available at https://osf.io/zgb5v/ upon publication. |

For manuscripts utilizing custom algorithms or software that are central to the research but not yet described in published literature, software must be made available to editors and reviewers. We strongly encourage code deposition in a community repository (e.g. GitHub). See the Nature Portfolio guidelines for submitting code & software for further information.

## Data

Policy information about availability of data

All manuscripts must include a data availability statement. This statement should provide the following information, where applicable:

- Accession codes, unique identifiers, or web links for publicly available datasets
- A description of any restrictions on data availability
- For clinical datasets or third party data, please ensure that the statement adheres to our policy

The data generated in this study will be available on the OSF at https://osf.io/zgb5v/ upon publication.

## Research involving human participants, their data, or biological material

Policy information about studies with human participants or human data. See also policy information about sex, gender (identity/presentation), and sexual orientation and race, ethnicity and racism.

| | |
|---|---|
| Reporting on sex and gender | We used the term 'gender' to refer to participants' self-reported gender. We reported key statistics regarding gender, such as the proportion of men and women in both young and older participants. |
| Reporting on race, ethnicity, or other socially relevant groupings | We did not collect data on race or ethnicity. |
| Population characteristics | See below. |
| Recruitment | We recruited 80 young participants and 81 older participants to take part in this study. Participants were recruited from university databases, social media, and the community for both age groups to make sure participants were matched as closely as possible. While our age groups were matched on years of education and IQ, the recruitment from university databases or issues regarding self-selection might mean that the levels of education and IQ in our sample are not completely representative of the general population. The self-selection bias could impact our results by meaning that the age-related differences observed in our study may only apply to people with levels of education and IQ within the range of our participants. However, controlling for these measures did not change our results. |
| Ethics oversight | University of Oxford Medical Sciences Interdivisional Research Ethics Committee. |

Note that full information on the approval of the study protocol must also be provided in the manuscript.

# Field-specific reporting

Please select the one below that is the best fit for your research. If you are not sure, read the appropriate sections before making your selection.

☐ Life sciences ☒ Behavioural & social sciences ☐ Ecological, evolutionary & environmental sciences

For a reference copy of the document with all sections, see nature.com/documents/nr-reporting-summary-flat.pdf

# Behavioural & social sciences study design

All studies must disclose on these points even when the disclosure is negative.

| | |
|---|---|
| Study description | The study analyses quantitative data, mainly from an experimental mixed design with a between-subject factor age group (young/older) and a within-subject factor other's preference (impulsive/patient) in the social discounting task. Additional measures are from self-report questionnaires and neuropsychological tests. |
| Research sample | 76 young (aged 18-36, mean=23.1, 45 females) and 78 older (aged 60-80, mean=70.0, 41 females) adults carefully matched on gender, years of education, and IQ were included in the main analysis (see below). The samples were not intended to be representative of any specific population. The sample age ranges were chosen to cover early adulthood and older adulthood. |
| Sampling strategy | The sampling strategy was self-selection as participants signed up by responding to the adverts circulated via university databases (including volunteers who are students and members of the community), social media, and local newspapers. We required the sample to consist of two age groups - young and older - matched on years of education and IQ. Therefore, inclusion in the sample was partially determined by whether a participant contributed toward this matching. This sample size gave us 87% power to detect a significant effect of interest, as determined through a simulation-based power analysis |
| Data collection | Data were collected on a computer for the social discounting and Apathy Motivation Index. A member of the research team was present for this data collection but did not observe the participant's responses while completing these measures. The Wechsler Test of Adult Reading (WTAR) was also administered by a member of the research team. The WTAR was recorded by the researcher on paper. Researchers could not be blind to the age-group condition but did not observe the participants completing the social discounting task. |
| Timing | 14th September 2016 - 27th August 2019. |
| Data exclusions | Four young and three older participants were excluded from all analyses due to: diagnosis of a neuropsychiatric disorder at the time of testing (one young participant); previous study of psychology (two young participants); potential risk for dementia (one older participant); and failure to complete the task (one young and two older participants). This left a final sample of 154 participants, 76 young participants (45 females aged 18-36, mean = 23.1) and 78 older participants (41 females aged 60-80, mean = 70.0). One participant from each age group was missing data on the self-report questionnaire measures and were excluded from the relevant analyses. In the final sample, eight young and four older participants had two agents with similar patient preferences. Data from these participants was excluded from all analyses involving the agent with impulsive preferences, as there was no available data. Similarly, four young and ten older participants in the final sample had two agents with similar impulsive preferences. Their data was also excluded from analyses involving the agent with patient preferences due to a lack of data. |

| | |
|---|---|
| Non-participation | No participants dropped out after starting the study. |
| Randomization | Groups were determined by age so naturally occurring. The groups were matched on gender, years of education, and IQ and we controlled for age-standardised intelligence in our control analyses. The factor other's preference was manipulated within-subjects but the order of the preferences (more impulsive vs more patient) was counterbalanced across participants. |

# Reporting for specific materials, systems and methods

We require information from authors about some types of materials, experimental systems and methods used in many studies. Here, indicate whether each material, system or method listed is relevant to your study. If you are not sure if a list item applies to your research, read the appropriate section before selecting a response.

## Materials & experimental systems

| n/a | Involved in the study |
|---|---|
| ☒ | ☐ Antibodies |
| ☒ | ☐ Eukaryotic cell lines |
| ☒ | ☐ Palaeontology and archaeology |
| ☒ | ☐ Animals and other organisms |
| ☒ | ☐ Clinical data |
| ☒ | ☐ Dual use research of concern |
| ☒ | ☐ Plants |

## Methods

| n/a | Involved in the study |
|---|---|
| ☒ | ☐ ChIP-seq |
| ☒ | ☐ Flow cytometry |
| ☒ | ☐ MRI-based neuroimaging |

# Plants

| | |
|---|---|
| Seed stocks | *Report on the source of all seed stocks or other plant material used. If applicable, state the seed stock centre and catalogue number. If plant specimens were collected from the field, describe the collection location, date and sampling procedures.* |
| Novel plant genotypes | *Describe the methods by which all novel plant genotypes were produced. This includes those generated by transgenic approaches, gene editing, chemical/radiation-based mutagenesis and hybridization. For transgenic lines, describe the transformation method, the number of independent lines analyzed and the generation upon which experiments were performed. For gene-edited lines, describe the editor used, the endogenous sequence targeted for editing, the targeting guide RNA sequence (if applicable) and how the editor was applied.* |
| Authentication | *Describe any authentication procedures for each seed stock used or novel genotype generated. Describe any experiments used to assess the effect of a mutation and, where applicable, how potential secondary effects (e.g. second site T-DNA insertions, mosiacism, off-target gene editing) were examined.* |
